# Preoperative Assessment of Patients Undergoing Elective Gastrointestinal Surgery: Does Body Mass Index Matter?

**DOI:** 10.1155/2017/4285204

**Published:** 2017-06-12

**Authors:** Sivesh K. Kamarajah, Mustafa Sowida, Amirul Adlan, Behrad Barmayehvar, Christina Reihill, Parvez Ellahee

**Affiliations:** ^1^College of Medical and Dental Sciences, University of Birmingham, Birmingham, UK; ^2^Pre-Operative Assessment Unit, Queen Elizabeth Hospital Birmingham, Birmingham, UK

## Abstract

**Background:**

At Queen Elizabeth Hospital Birmingham (QEHB), no specific protocol to stratify patients by body mass index (BMI) exists. This study sought to evaluate outcomes following gastrointestinal surgery.

**Methods:**

Patients undergoing gastrointestinal surgery attending preassessment screening clinic (PAS) from August to September 2016 at the QEHB were identified. Primary outcome was postoperative complications. Secondary outcomes were major complications and 30-day readmission rates.

**Results:**

Of 368 patients preassessed, 31% (116/368) were overweight and 35% (130/368) were obese. Median age was 57 (range: 17–93). There was no difference of BMI between the low risk and high risk clinics. Patients in high risk clinic had significantly higher rates of comorbidities, major surgical grades, and malignancy as the indication for surgery. Overall complication rates were 14% (52/368), with 3% (10/368) having major complications (Clavien-Dindo Grades III-IV). Whilst BMI was associated with comorbidity (*p* = 0.03) and ASA grade (*p* < 0.001), it was not associated with worse outcomes. Patients attending high risk clinic had significantly higher rates of complications.

**Conclusions:**

Surgery grade was found to be an independent risk factor of complication rates. Use of BMI as an independent factor for preassessment level is not justified from our cohort.

## 1. Introduction

The incidence of obesity is rising rapidly across developed countries, with current prevalence in the United States (35.7%) and United Kingdom (26.1%) expected to double by 2050 [1, 2]. Estimates predict that up to 66% of patients undergoing surgery in the UK were overweight. Current evidence conflicts regarding the impact of obesity on postoperative complications following major surgery. Multicentre studies in specific patient groups from Japan, Denmark, Switzerland, and the USA have associated obesity with worse or neutral short term postoperative outcomes [3–7].

Despite this, limited evidence exists surrounding the benefit of preoperative management of obese patients in dedicated high risk preassessment clinics and its implications on postoperative outcomes such as complications and length of hospital stay. The recently published guidelines by the Association of Anaesthetists of Great Britain & Ireland (AAGBI) recommended that all obese patients receive preoperative assessment by an anaesthetist in high risk clinics [8]. However, clinical evidence behind this recommendation is lacking and instead this is largely based on expert opinion. Since the variable of interest was body mass index (BMI), randomised trials assigning patients to preassessment clinics are not possible.

To build on the limited literature and offer further clarification on the need for preassessment stratification by BMI, this prospective study sought to evaluate the impact of BMI on postoperative outcomes in patients undergoing preoperative assessment for gastrointestinal surgery.

## 2. Methods

### 2.1. Inclusion and Exclusion Criteria

This prospective study identified patients attending preassessment screening clinic (PAS) for minor to major gastrointestinal surgery attending from August 2016 to September 2016 at the Queen Elizabeth Hospital Birmingham (QEHB). Consecutive, adult patients (≥18 years) with a BMI > 30 kg/m^2^ undergoing gastrointestinal or hepatobiliary surgery were included in the study. Eligible procedures were those involving surgery on any part of the gastrointestinal tract or biliary tree, involving a hospital admission with an overnight stay. Patients undergoing day-case urological, gynaecological, vascular, or transplant procedures were excluded. This study was registered and approved by the local audit department. Patients' medical records were reviewed and the data were extracted on to a uniform database (Microsoft® Excel 2010) that was designed to include all relevant details pertinent to this study.

### 2.2. Preassessment Clinics

At QEHB, all patients undergoing surgical procedures were referred by the surgeon to dedicated preassessment clinics according to surgery grade and comorbidities. Clinics were divided into low risk and high risk; numerically these corresponded to levels 1 and 2A and 2B and 3, respectively. Low risk clinics were led and delivered by trained preassessment nurses whereas high risk clinics were led and delivered by more experienced nursing staff and consultant anaesthetists. As no systematic method for stratifying risk was in place, each patient was assigned a PAS clinic based on a preliminary individual risk assessment by the referring surgeon.

### 2.3. Main Explanatory Variable

The main explanatory variable was preoperative BMI, assessed during attendance at PAS clinic. BMI was calculated as weight (in kilograms) divided by height (in metres) squared. Given the primary aim of this study which was to assess the impact of being overweight or obese, patients were stratified by BMI into groups defined by the World Health Organization (WHO): normal weight (BMI 18.5–24.9 kg/m^2^), overweight (BMI 25.0–29.9 kg/m^2^), and obese (BMI ≥ 30.0 kg/m^2^).

### 2.4. Explanatory Variables

Explanatory variables were collected to provide a risk‐adjusted estimates. Variables were predefined and selected based on clinical plausibility. Comorbidities were measured using the American Society of Anesthesiologists (ASA) fitness grade, a standardised metric for disease severity and a reliable method for the measurement of postoperative mortality and complications [9]. Grade of surgery is a category that indicates a combination of complexity and amount of tissue injury in the surgical procedure. Exact definitions used were similar to those in a recent publication from the European Surgical Outcomes Study [10]. Surgical approach was defined as open, laparoscopic, or endoscopic/ultrasound (for minor surgical grade only).

### 2.5. Outcome Measures

The primary outcome measure was 30-day complications as defined by the Clavien-Dindo classification system [11]. Secondary outcome measures were major complications, 30-day readmission rates, and postoperative care setting.

### 2.6. Statistical Analysis

This study was powered to detect a minimum significant difference between normal weight, overweight, and obese patients. A minimum of 356 patients were needed to provide 80% power (*α* = 0.05). Baseline characteristics were compared between groups using Pearson chi-square (*χ*^2^) test for categorical variables and Mann–Whitney *U* test for continuous variables. Multivariate logistic regression was used to determine the strength association between risk factors for postoperative complications. Models built included age, sex, ethnicity, grade of surgery, presence of specific comorbid diseases, surgical specialty, and surgical approach. Results are presented as hazard ratios (HRs) with 95 per cent confidence interval (CI_95%_). In all analyses, a *p* value of < 0.05 was maintained as statistically significant. All data analyses were performed using IBM SPSS Statistics Version 22.0.

## 3. Results

### 3.1. Baseline Demographics

During this study period, 376 patients were identified through PAS and only 2% (8/376) had cancellations for their procedure and hence were not included in further analysis. Baseline demographics of patients who had preassessment and then went on to have surgery are presented in Table 1. Of these 368 patients, 33% (122/368) were of normal weight, 31% (116/368) were overweight, and 35% (130/368) were obese. Median age is 57 (range: 17–93) and 55% (204/368) were male.

There was no significant difference in surgery grade between the BMI groups (*p* = 0.185). However, 48% of obese patients had >2 comorbidities as compared to 30% and 40% in overweight and normal BMI patients (*p* = 0.034), respectively. Furthermore 26% of obese patients had ASA grades 3 or 4 as compared to 16% and 13% in the overweight and normal BMI groups (*p* < 0.001), respectively.

### 3.2. Surgery Types

The most common indications for surgery were malignancy accounting for 25% of cases followed by hernia (23%) and cholecystitis (18%). In normal and overweight patients, malignancy was the most common indication for surgery followed by hernia repair and cholecystitis. In obese patients, both malignancy (22%) and cholecystitis (22%) were the most common indications followed by hernia repair. However, appendicitis was the least most common indication for surgery in this cohort and across different BMI groups. The indications for surgery in this cohort are presented in Table 2.

### 3.3. Postoperative Outcomes

Overall complication rates were 14% (52/368), with 3% (10/368) having major complications (Grades III-IV). Postoperative outcomes are presented in Table 3. Rates of overall postoperative complications and major complications were similar across the BMI categories (*p* = 0.854, *p* = 0.950, respectively). There was no significant difference in 30-day readmission rates (*p* = 0.827).

Multivariate logistic regression was produced to identify the impact of BMI on postoperative outcomes. In adjusted models accounting for covariates presented in Table 4, there was no impact of BMI on overall complications (overweight, HR: 1.34 (CI_95%_: 0.55–3.27), *p* = 0.52; obese, HR: 1.32 (CI_95%_: 0.54–3.24), *p* = 0.55). When stratified by surgical grade, there was no significant impact of BMI on postoperative complications.

### 3.4. Characteristic by Preassessment Clinic

In this cohort, 28% of patients were seen in the high risk clinic (levels 2B and 3). There were significantly more patients in the high risk clinic with >2 comorbidities than the low risk clinic (54% versus 34%, *p* < 0.001). Sixty-six percent (66%) of patients seen in the high risk clinic underwent a major surgical procedure (*p* < 0.001) as compared to only 8% in the low risk clinic. There was a significantly higher rate of open procedures (57% versus 29%, *p* < 0.001) and surgeries for malignant indication (51% versus 13%, *p* < 0.001) in the high risk clinic as compared to the low risk clinic. No significant differences in ASA grade or BMI were observed between the groups.

### 3.5. Postoperative Outcomes by Preassessment Clinic

Following surgery, 88% of patients from the high risk clinic were sent to either ward or enhanced care for recovery. This contrasts with only 20% from the low risk clinic. There were significantly higher rates of overall complications (31% versus 8%, *p* < 0.001) and major complications (7% versus 1%, *p* = 0.003) in the high risk clinic as compared to the low risk clinic. There were also higher 30-day readmission rates in patients seen in high risk clinics as compared to those seen in low risk clinics (14% versus 4%, *p* = 0.001).

### 3.6. Cancellation Rates

Eight patients had cancelled surgery, of which 6 were from the nurse-led low risk clinic. Of these 6 patients, 2 patients had their surgery deferred due to poor diabetic control, 1 refused surgery on the day of procedure, and 1 was admitted for abdominal pain before surgery. One patient scheduled for a Nissen fundoplication felt unsuitable to proceed for surgery by the surgeon on the day of procedure due to high BMI (38.2 kg/m^2^).

## 4. Discussion

This study aimed at assessing the need for BMI as a factor for stratifying patients into PAS clinics. Results of this study demonstrate that surgical grade rather than BMI is an independent risk factor for overall complications. When the cohort was stratified by surgical grade, BMI remains a nonsignificant factor in overall complications.

Currently, there is conflicting evidence as to the importance of BMI as a risk factor for postoperative complications. Evidence suggests that obese patients undergoing surgery for a malignant indication are at an increased risk of complications [12, 13]. However, this increased risk is not observed in patients undergoing surgery for a benign indication. Such findings demonstrate that obese patients undergoing surgery for a malignant indication may need to be seen in high risk clinics. A systematic review of patients undergoing laparoscopic colorectal surgery concluded that BMI was not a predictor of increased postoperative complication rates or length of hospital stay [14].

Despite these papers demonstrating varying evidences for the impact of BMI, preassessment services do not consider BMI as a risk factor during triage. Current literature on the approach to preassessment in the context of BMI and obesity is limited, and although advocating that a nurse-led and consultant/specialist-led PAS clinic is feasible, this service remains largely undefined considering the range of surgical specialties [15–17]. At QEHB, this two-tiered screening clinic was recently introduced to allow assessment of patients by risk groups based on their comorbidities and ASA grade. This service allows risk assessment of patients for their comorbidities such as diabetes and cardiovascular disease prior to surgery. This was achieved by means of referral to the medical specialty of concern for review and advice prior to surgery. This aimed at reducing cancellations on the day of surgery and postoperative complications.

One of the limitations to this study is that data was derived from a single centre. Our results require validation from multiple centres using a similar approach to preassessment of obese patients. Nonetheless, this study remains the first in the literature reporting outcomes in preassessment services for patients undergoing gastrointestinal surgery. Furthermore, this study accounts for varying grade of surgical procedure making it difficult to assess need for BMI for triaging. Subgroup analysis demonstrated that BMI did not impact outcomes when stratified by surgical grade. This study only included patients undergoing elective procedures and hence results may not be comparable to previous studies including both elective and emergency procedures.

## 5. Conclusion

In summary, specific triaging for BMI may not be required for preassessment clinics in patients undergoing gastrointestinal surgeries. However, future prospective studies should aim to confirm these findings and help establish the need for consideration of BMI in planning patient triage and perioperative management.

## Figures and Tables

**Table 1:** Baseline characteristics of patients by body mass index (BMI).

Variable	Normal, *n* (%)	Overweight, *n* (%)	Obese, *n* (%)	*p* value
Age (range)	60 (17–92)	52 (19–83)	58 (21–87)	
Sex				
Male	74 (61)	74 (64)	56 (43)	0.002^*∗*^
Female	48 (39)	42 (36)	74 (57)	
Comorbidity				
0	20 (16)	25 (22)	14 (11)	0.034^*∗*^
0-1	53 (43)	56 (48)	53 (41)	
>2	49 (40)	35 (30)	63 (48)	
ASA grade				
1	19 (16)	26 (22)	0 (0)	<0.001^*∗*^
2	87 (71)	72 (62)	95 (74)	
3	15 (12)	17 (15)	32 (25)	
4	1 (1)	1 (1)	1 (1)	
Surgical grade				
Minor	40 (33)	44 (38)	39 (30)	0.185
Intermediate	50 (41)	40 (35)	65 (50)	
Major	32 (26)	31 (27)	26 (20)	
Surgical type				
Upper GI	37 (30)	22 (19)	25 (19)	0.042^*∗*^
Lower GI	35 (29)	51 (44)	45 (35)	
HPB	50 (41)	42 (37)	60 (46)	
Indication				
Benign	92 (75)	86 (74)	102 (78)	0.713
Malignant	30 (25)	30 (26)	28 (22)	
Operative approach				
Endoscopic/ultrasound	37 (30)	43 (37)	42 (32)	0.104
Laparoscopic	35 (29)	27 (23)	49 (38)	
Open	50 (41)	46 (40)	39 (30)	
Smoking status				
Current	60 (49)	66 (57)	68 (53)	0.224
Ex-smoker	30 (25)	30 (26)	24 (19)	
Never	32 (26)	18 (16)	35 (27)	
Unknown	0 (0)	2 (2)	2 (2)	

Upper Gastrointestinal Surgery (Upper GI), Lower Gastrointestinal Surgery (Lower GI), and hepatobiliary surgery (HPB). *∗* means that these variables are significant when tested using chi-square.

**Table 2:** Surgical indications.

Indications	Normal, *n* (%)	Overweight, *n* (%)	Obese, *n* (%)
Malignant	32 (26)	30 (26)	29 (22)
Hernia	30 (25)	27 (23)	27 (21)
Cholecystitis	20 (16)	18 (16)	29 (22)
All other indications	8 (6)	12 (11)	12 (9)
Diagnostic chronic liver disease	7 (6)	5 (4)	8 (6)
Anal fistula	6 (5)	11 (9)	6 (5)
Haemorrhoids	5 (4)	5 (4)	7 (5)
Inflammatory bowel disease	5 (4)	2 (2)	0 (0)
Other liver or pancreatic disease	4 (3)	1 (1)	6 (5)
Gastrooesophageal reflux	3 (2)	2 (2)	2 (2)
Pancreatitis	1 (1)	1 (1)	0 (0)
Faecal incontinence	1 (1)	1 (1)	3 (2)
Appendicitis	0 (0)	0 (0)	1 (1)
Diverticular disease	0 (0)	1 (1)	0 (0)

**Table 3:** Postoperative outcomes by BMI.

Variable	Normal, *n* (%)	Overweight, *n* (%)	Obese, *n* (%)	*p* value
Postoperative complications				
No	106 (87)	98 (84)	112 (86)	0.854
Yes	16 (13)	18 (16)	18 (15)	
Major Complications				
No	119 (98)	113 (97)	126 (97)	0.950
Yes	3 (2)	3 (3)	4 (3)	
30-day readmission rate				
No	113 (94)	108 (93)	118 (92)	0.827
Yes	7 (6)	8 (7)	10 (8)	
Unplanned CCA				
No	118 (97)	112 (97)	125 (97)	0.716
Yes	4 (3)	3 (3)	4 (3)	

Critical care admission (CCA).

**Table 4:** Multivariate model.

Variables	HR (CI_95%_)	*p* value
Age	1.02 (1.00–1.04)	0.123
Sex		
Male	REF	—
Female	2.12 (1.01–4.44)	0.047
Comorbidity		
0	REF	—
0-1	1.82 (0.43–7.74)	0.419
>2	1.57 (0.33–7.46)	0.571
ASA grade		
1	REF	—
2	0.58 (0.14–2.43)	0.456
3	1.15 (0.23–5.65)	0.865
4	—	0.999
Surgical grade		
Minor	REF	—
Intermediate	1.93 (0.25–14.89)	0.529
Major	21.64 (2.24–209.49)	0.008
Surgical type		
Upper GI	REF	—
Lower GI	0.71 (0.26–1.93)	0.504
HPB	0.54 (0.21–1.40)	0.206
Indication		
Benign	REF	—
Malignant	0.88 (0.36–2.16)	0.782
Operative approach		
Endoscopic/ultrasound	REF	—
Laparoscopic	0.95 (0.12–7.66)	0.957
Open	1.30 (0.16–10.64)	0.807
PAS clinic		
Low risk	REF	—
High risk	0.98 (0.38–2.56)	0.971
BMI		
Normal	REF	—
Overweight	1.34 (0.55–3.27)	0.519
Obese	1.32 (0.54–3.23)	0.547

Upper Gastrointestinal Surgery (Upper GI), Lower Gastrointestinal Surgery (Lower GI), hepatobiliary surgery (HPB), preassessment service (PAS), and body mass index (BMI).
